# Degradation and Detoxification of Aflatoxin B1 by Tea-Derived *Aspergillus niger* RAF106

**DOI:** 10.3390/toxins12120777

**Published:** 2020-12-06

**Authors:** Qian’an Fang, Minru Du, Jianwen Chen, Tong Liu, Yong Zheng, Zhenlin Liao, Qingping Zhong, Li Wang, Xiang Fang, Jie Wang

**Affiliations:** 1 Guangdong Provincial Key Laboratory of Food Quality and Safety, College of Food Science, South China Agricultural University, Guangzhou 510642, China; twingofang@163.com (Q.F.); duminru2016@163.com (M.D.); cjw905368952@126.com (J.C.); Pluto0523@126.com (T.L.); zhengyong@qianweish.com (Y.Z.); larryliao@scau.edu.cn (Z.L.); zhongqp@scau.edu.cn (Q.Z.); wangli_scau@scau.edu.cn (L.W.); 2 Lingnan Guangdong Laboratory of Modern Agriculture, Guangzhou 510642, China; 3 Guangdong Open Laboratory of Applied Microbiology, Guangdong Provincial Key Laboratory of Microbial Culture Collection and Application, State Key Laboratory of Applied Microbiology Southern China, Guangdong Institute of Microbiology, Guangdong Academy of Sciences, Guangzhou 510070, China

**Keywords:** aflatoxin B1, *Aspergillus niger*, intracellular extracts, Ames test, genome sequencing

## Abstract

Microbial degradation is an effective and attractive method for eliminating aflatoxin B1 (AFB1), which is severely toxic to humans and animals. In this study, *Aspergillus niger* RAF106 could effectively degrade AFB1 when cultivated in Sabouraud dextrose broth (SDB) with contents of AFB1 ranging from 0.1 to 4 μg/mL. Treatment with yeast extract as a nitrogen source stimulated the degradation, but treatment with NaNO_3_ and NaNO_2_ as nitrogen sources and lactose and sucrose as carbon sources suppressed the degradation. Moreover, *A. niger* RAF106 still degraded AFB1 at initial pH values that ranged from 4 to 10 and at cultivation temperatures that ranged from 25 to 45 °C. In addition, intracellular enzymes or proteins with excellent thermotolerance were verified as being able to degrade AFB1 into metabolites with low or no mutagenicity. Furthermore, genomic sequence analysis indicated that the fungus was considered to be safe owing to the absence of virulence genes and the gene clusters for the synthesis of mycotoxins. These results indicate that *A. niger* RAF106 and its intracellular enzymes or proteins have a promising potential to be applied commercially in the processing and industry of food and feed to detoxify AFB1.

## 1. Introduction

Mycotoxins, such as aflatoxins, ochratoxin A, fumonisins, deoxynivalenol, and zearalenone, are toxic metabolites that are primarily produced by fungi, including *Aspergillus*, *Penicillium*, and *Fusarium*, and have contaminated approximately 60–80% of the food and feed around the world [1,2]. For example, the incidence and maximum level in raw cereal grains were 55% and 1642 µg/kg for aflatoxins based on the global occurrence data reported during the past 10 years [1]. Among them, aflatoxin B1 (AFB1), a bisfuranocoumarin derivative, is the most toxic contaminant and causes carcinogenic, teratogenic, hepatotoxic, and immunosuppressive effects in humans and many types of animals, including poultry, trout, cattle, and rats, with different incidences across species, gender, and age [3,4,5]. Therefore, it poses a serious threat to livestock productivity and human health. The key sites in AFB1 regarding its toxicity, mutagenicity, and carcinogenesis are the lactone ring in the common coumarin structure and the double bond of the terminal furan rings [6,7]. In addition, owing to its high liposolubility, AFB1 can easily enter the bloodstream and reach organs, particularly the liver, and be metabolized to aflatoxin M1 (AFM1) there. AFM1 can appear in milk, and thus dairy products, and exhibit carcinogenic and immunosuppressive effects similar to that of AFB1, even if with a less potent effect [8,9]. Therefore, the contamination of AFB1 in food and feed results in enormous economic losses and public health and safety problems [10,11]. AFB1-related health problems tend to be the most severe in developing nations, and approximately 4.5 billion people are chronically exposed owing to the lack of regulation of AFB1 [12]. Considering the potential hazards to animal and human health, there is an urgent need to lower the content of AFB1 to the established permissible levels in food and feed, which vary from 0 to 50 µg/kg in different countries [13].

Several ways, including physical, chemical, and biological methods, have been proposed to eliminate AFB1 and prevent agricultural products from AFB1 contamination. Compared with physical and chemical methods, microbial degradation is receiving much attention owing to its advantages, such as the mild reactions, minimal loss of product qualities, eco-friendly nature, and feasible processes when applied in an industrial setting [14,15]. Over the past several decades, it has been reported that many bacteria and fungi can detoxify AFB1 to less toxic metabolites. For example, AFB1 was converted into AFB2a and AFD1 by *Lactobacillus delbrueckii* [16]; AFD1, AFD2, and AFD3 by *Pseudomonas putida* [17]; AFD1 and AFD2 by *Rhodococcus erythropolis* [18]; AFB1-8,9-dihydrodiol by *Phanerochaete sordida* YK-624 [19]; aflatoxicol by *Eurotium herbariorum*, *Rhizopus* spp., *Mucor alternans*, and *Trichoderma viride* [20]. Although these fungi and bacteria can detoxify AFB1, few strains have been developed into a commercial product for the biological prevention of aflatoxin contamination because of the narrow working temperature range, unsuitability for the processing environment, and the safety of these microorganisms [21,22]. Therefore, it is still worth exploring safe microorganisms that are suitable for food and feed processing and can degrade AFB1 to less toxic metabolites with excellent degradation efficiency over wide temperature and pH ranges.

*Aspergillus niger*, a filamentous ascomycete fungus that is generally recognized as safe (GRAS) by the U.S. Food and Drug Administration (FDA), has been widely applied in the fermentation industry and food processing, including the production of various organic acids, enzymes, and bioactive compounds, as well as the improvement of the sensory characteristics of fermented foods [23,24,25]. For example, in post-fermented Pu-erh tea, *A. niger* is one of the dominant strains and produces various enzymes that catalyze the metabolism of chemical compounds, such as converting tea polyphenols to bioactive theabrownins and producing the main flavor characteristics of Pu-erh tea [25]. Recently, *A. niger* ND-1 isolated from feed ingredients and *A. niger* FS-Z1 have been reported to be capable of degrading AFB1 [26,27]. However, the highest degradation rate of AFB1 in wild-type *A. niger* is 58.52% after 48 h of incubation, and with the exception of aflatoxicol, the metabolites from AFB1 degradation mediated by *A. niger* and their toxicity remain unknown [26,27,28]. Additionally, it has been reported that some *A. niger* strains produce mycotoxins, such as ochratoxin and fumonisin [29,30], but it was still unknown whether the AFB1-degrading *A. niger* strains produce mycotoxins.

In this study, we found that *A. niger* RAF106, a catechin-transforming fungus isolated from Pu-erh tea [31], could degrade AFB1 by 88.59% after 72 h. The initial part of this study that focused on improving the degradation rates of AFB1 examined the optimal conditions by assessing the effects of the AFB1 concentrations, nutrients, initial pH, and temperatures on the degradation of AFB1. Second, to illuminate the characteristics of AFB1 degradation mediated by *A. niger* RAF106, the active ingredient to degrade AFB1 and the potential metabolites were investigated in optimal conditions. Lastly, the mutagenic potential of the degradation metabolites was also evaluated, and the genome of *A. niger* RAF106 was sequenced to assess whether the strain is a potential atoxigenic strain. These results will be helpful in developing safe and effective AFB1-degrading agents that are associated with *A. niger* RAF106, which can be commercially used in the process and manufacture of food and feed. 

## 2. Results

### 2.1. AFB1 Degradation by A. niger RAF106 and the Effect of AFB1 on Fungal Development 

**Figure 1:**
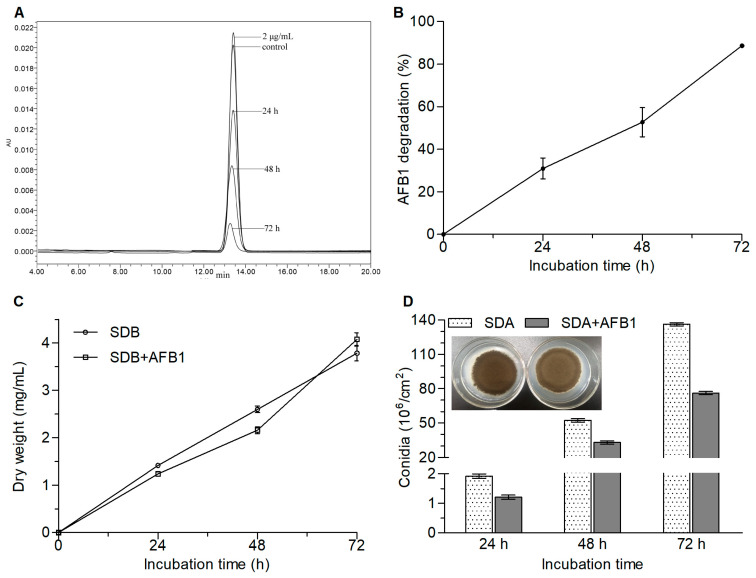
Changes in the residual AFB1 and fungal development when *A. niger* RAF106 was co-cultivated with 2 μg/mL AFB1 for 72 h at 30 °C. (**A**) High-performance liquid chromatography (HPLC) chromatogram of 2 μg/mL AFB1 and extracted AFB1 from the AFB1-degrading cultures mediated by *A. niger* RAF106 for different incubation times. The concentration of AFB1 incubated in Sabouraud dextrose broth (SDB) with shaking was used as control. (**B**) The percentages of AFB1 degradation for different incubation times. (**C**) Dry weight of *A. niger* RAF106 cultivated in SDB and SDB with AFB1 for different incubation times. (**D**) The colony and conidial yields of *A. niger* RAR106 grown on the Sabouraud dextrose agar (SDA) and SDA supplemented with AFB1 for different incubation times.

There was no significant variation in the concentration (Tukey’s honestly significant difference (HSD), *p* > 0.05) when AFB1 was incubated in Sabouraud dextrose broth (SDB) with shaking for 72 h at 30 °C (data not shown). However, the chromatographic peak area of AFB1 decreased as the incubation time increased (Figure 1A) when incubated with *A. niger* RAF106. The percentage of AFB1 degradation was 30.99%, 52.71%, and 88.59% at the incubation times of 24, 48, and 72 h, respectively (Figure 1B), suggesting that *A. niger* RAF106 could degrade AFB1 in a time-dependent manner. Moreover, compared with those of the cultures with no AFB1, the biomass of *A. niger* RAF106 at 24 and 48 h was slightly reduced by 12.78–16.94% (Tukey’s HSD, *p* < 0.05), but the biomass at 72 h showed no significant changes (Figure 1C), suggesting that AFB1 did not inhibit the hyphal growth of *A. niger* RAF106 over 72 h of incubation. However, when incubated with *A. niger* RAF106 on Sabouraud dextrose agar (SDA) plates, AFB1 caused some defects in the production of the black conidia, although no obvious change was observed in the diameters of the fungal colonies (Figure 1D). As shown in Figure 1D, the presence of AFB1 caused a 33.22–45.24% decrease in the conidial yields of *A. niger* RAF106 over 72 h of cultivation (Tukey’s HSD, *p* < 0.05). These results demonstrated that the hyphal growth of *A. niger* RAF106 was almost unaffected during the degradation of AFB1, although a reduction in conidiation was observed.

### 2.2. Effects of the Fermentation Conditions on AFB1 Degradation 

**Figure 2:**
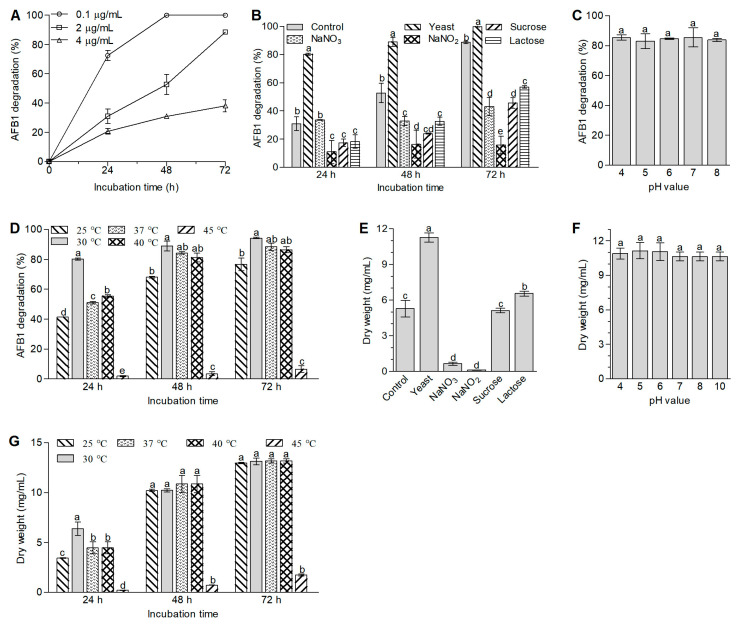
Effect of AFB1 concentrations (**A**), nutrients (**B**), initial pH value (**C**), and incubation temperature (**D**) on the degradation of AFB1 by *A. niger* RAF106 over 72 h of incubation and fungal biomass (**E**–**G**) after *A. niger* RAF106 was cultivated for 72 h. The effects of nutrients were determined by replacing tryptone in the native SDB with yeast extract, NaNO_3_, or NaNO_2_ as a single nitrogen source and replacing glucose with lactose or sucrose as a single carbon source. The effects of the initial pH values and temperatures were investigated in the modified SDB with yeast extract. Different lowercase letters on the bars of each group indicate significant differences between the treatments (Tukey’s HSD, *p* < 0.05).

To accelerate the degradation of AFB1, the effects of the AFB1 concentrations, nutrients, initial pH values, and temperatures on the degradation of the compound were investigated for *A. niger* RAF106. In terms of the AFB1 concentrations, we found that the degradation rate decreased with an increase in the initial concentrations of AFB1 (Figure 2A). For example, in a comparison of the degradation activity at 24 h, compared with the estimate of 72.50% in the group with 0.1 μg/mL AFB1, the percentage of AFB1 degradation decreased by 57.26% in the group with 2 μg/mL AFB1 (Student’s *t*_4_ = 16.70, *p* = 0.0001) and 71.45% in the group with 4 μg/mL AFB1 (Student’s *t*_4_ = 31.53, *p* = 0.0001). Considering the limit of quantitation (LOQ) and the maximum level in raw cereal grains, 2 μg/mL AFB1 was chosen to conduct the following studies.

With respect to the effects of nutrients on the AFB1 degradation, it was found that the use of yeast extract as the nitrogen source accelerated the degradation of AFB1 by 12.68–158.90%, but NaNO_3_ and NaNO_2_ slowed the degradation by 37.67–82.15% compared with that in SDB with tryptone as the nitrogen source (native SDB) over 72 h of incubation (Figure 2B). When *A. niger* RAF106 was cultivated with AFB1 in modified SDB with yeast extract, the percentage of AFB1 degradation reached 80% at 24 h of fermentation (Figure 2B). In terms of carbon sources, both sucrose and lactose inhibited the degradation of AFB1 by 35.60–54.48% compared with that in SDB with glucose as the carbon source over 72 h of cultivation (Figure 2B).

With regard to the influence of the initial pH on the degradation of AFB1 in *A. niger* RAF106, we found that the degradation mediated by *A. niger* RAF106 was unaffected by the initial pH values. For example, there was no noticeable difference on the degradation of AFB1 between different initial pH values from 4 to 8 after 48 h (*F*_4,14_ = 0.49, *p* = 0.74; Figure 2C). When the initial pH value reached 10, the content of AFB1 was degraded to less than the LOQ value in the control group (data not shown).

Additionally, the degradation of AFB1 was affected by the fermentation temperature (Figure 2D). Compared with those at 30 °C, the percentages of AFB1 degradation decreased by 18.75–48.14% at 25 °C and by more than 90% at 45 °C over 72 h of incubation (Figure 2D). However, the percentages of AFB1 degradation at 37 °C and 40 °C were nearly identical to those at 30 °C when the incubation time lasted 48 h and 72 h, even though the percentages were inhibited by 30.81–36.29% after the first 24 h of incubation (Figure 2D).

Moreover, to assess whether the changes in the degradation of AFB1 caused by different fermentation conditions were dependent on fungal growth, the effects of nutrients, initial pH values, and temperatures on fungal growth were investigated in *A. niger* RAF106. As shown in Figure 2E, the presence of yeast extract or lactose promoted the fungal growth by 113.21% and 23.93%, respectively, but the presence of NaNO_3_ or NaNO_2_ inhibited the fungal growth by 87.60% and 97.89%, respectively, compared with the growth in the native SDB. However, the fungal growth in the presence of sucrose was similar to that in the native SDB (Student’s *t*_4_ = 0.53, *p* = 0.63). Among the different initial pH values that ranged from 4 to 10, there were no obvious changes in fungal growth (*F*_5,17_ = 1.11, *p* = 0.40; Figure 2F). In terms of temperature, compared with those at 30 °C, the fungal biomass was inhibited by 86.85–96.79% at 45 °C over 72 h of incubation (Figure 2G). However, the amounts of fungal biomass at 25 °C, 37 °C, and 40 °C were nearly identical to that at 30 °C when the incubation time lasted 48 h and 72 h, even though the biomass was inhibited by 30.26–46.16% after the first 24 h of incubation (Figure 2G). A comparison of the changes in the degradation of AFB1 and fungal biomass caused by fermentation conditions suggested that there was a correlation between AFB1 degradation and fungal growth, but the AFB1 degradation did not completely depend on fungal growth in *A. niger* RAF106.

These changes suggested that *A. niger* RAF106 could be a potential AFB1-degrading strain that can be used in various environments with AFB1 contents ranging from 0.1 to 4 μg/mL, pH values from 4 to 10, and temperatures from 25 to 45 °C.

### 2.3. AFB1 Degradation by Extracellular and Intracellular Extracts and Dead Cells of A. niger RAF106

**Figure 3:**
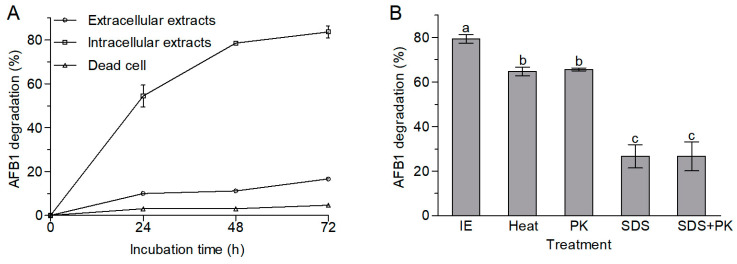
AFB1 degradation among the diverse cell components from *A. niger* RAF106. (**A**) Changes in the percentages of AFB1 degradation by extracellular extracts, intracellular extracts, and dead cells during 72 h of incubation with 2 μg/mL AFB1 at 30 °C. (**B**) Effects of heat, proteinase K (PK), sodium dodecyl sulfate (SDS), and SDS plus proteinase K on the degradation of AFB1 mediated by intracellular extracts (IE). After being treated with heat, proteinase K, SDS, or SDS plus proteinase K, the intracellular extracts were incubated with 2 μg/mL AFB1 at 30 °C for 48 h. Different lowercase letters on the bars of each group indicate significant differences between the treatments (Tukey’s HSD, *p* < 0.05).

The AFB1 degradation activity of intracellular extracts from *A. niger* RAF106 was significantly stronger than that of the extracellular extracts and dead cells (Tukey’s HSD, *p* < 0.05). The contents of AFB1 were relatively stable in the dead cells and decreased slightly by 10.00–16.69% in the extracellular extracts over the 72 h incubation period, but the intracellular extracts could degrade AFB1 by 54.44% over 24 h of incubation, 78.54% for 48 h of incubation, and 83.64% for 72 h of incubation (Figure 3A). These findings demonstrated that intracellular extracts rather than extracellular extracts and adsorption played significant roles in the degradation of AFB1 mediated by *A. niger* RAF106.

Moreover, the AFB1-degrading ability of the intracellular extracts declined significantly when the intracellular extracts were preconditioned with proteinase K and sodium dodecyl sulfate (SDS) (*F*_3,11_ = 240.33, *p* = 0.0001). The percentage of AFB1 degradation decreased from 79.35% to 65.60% upon treatment with proteinase K, and to 26.61% upon treatment with SDS or SDS plus proteinase K (Figure 3B). In addition, the intracellular extracts that were boiled for 20 min still degraded AFB1 by 64.68% (Figure 3B). These results strongly suggested that heat-stable intracellular enzymes or proteins might be the active ingredients for the degradation of AFB1 in *A. niger* RAF106.

### 2.4. The Ames Test for Mutagenicity

**Figure 4:**
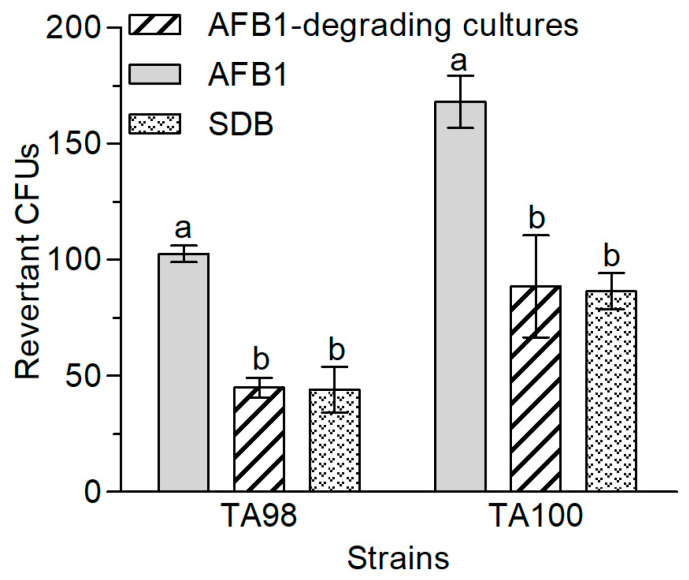
Reduction of AFB1 mutagenic effects by *A. niger* RAF106. Differences in revertant colony-forming units (CFUs) of *S. typhimurium* TA98 and TA100 between different groups. AFB1 group means extracts from the media supplemented with 20 μg AFB1. The group of AFB1-degrading cultures refers to the culture extracts from the supernatant of the 72 h co-incubation of 20 μg AFB1 and *A. niger* RAF106. The SDB group refers to extracts from the SDB media. Different lowercase letters on the bars of each group indicate significant differences between the treatments (Tukey’s HSD, *p* < 0.05).

For the purpose of detoxifying AFB1 to less toxic metabolites, it is essential to assess the toxicity of the degradation products. In this study, the Ames test was used to measure the mutagenicity of the degradation products from AFB1 mediated by *A. niger* RAF106. Compared with the AFB1 group (positive control), the revertant colony-forming units (CFUs) in the AFB1-degrading medium extracts noticeably decreased by 56.10% for *Salmonella typhimurium* TA98 (Student’s *t*_4_ = 25.51, *p* = 0.0001) and by 47.32% for *S. typhimurium* TA100 (Student’s *t*_4_ = 7.91, *p* = 0.0014) (Figure 4), which were equivalent to that in the SDB group (negative control). The decreases in mutagenicity suggested that *A. niger* RAF106 might detoxify AFB1 into less toxic or non-toxic compounds.

### 2.5. Analysis of the AFB1 Degradation Products

**Figure 5:**
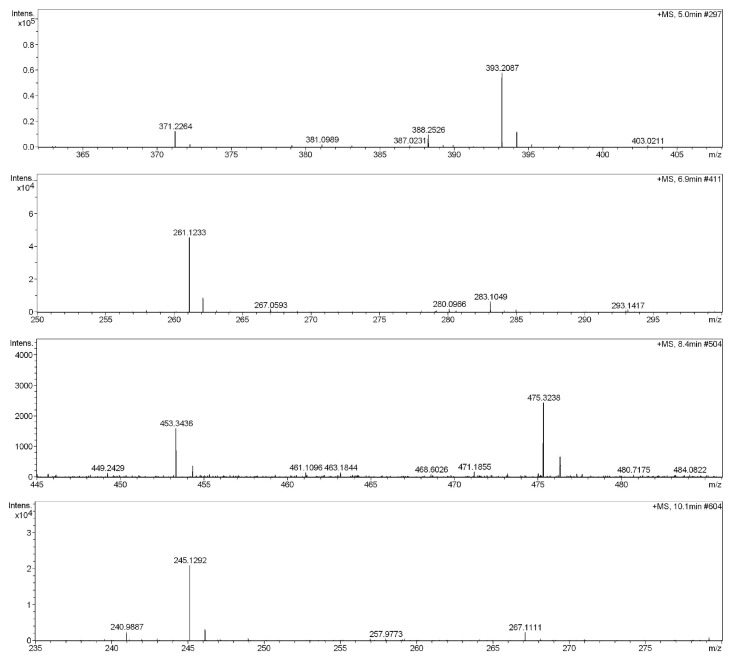
Mass spectra of degradation products for AFB1 by *A. niger* RAF106.

Compared with AFB1 and the metabolites produced by *A. niger* RAF106 in SDB, four new peaks appeared in the AFB1-degrading cultures after 72 h of incubation (Figure 5), according to the high-performance liquid chromatography–quadrupole time-of-flight mass spectrometry (HPLC–Q-TOF-MS) data, and they corresponded with the molecular formulae C_16_H_34_O_9_ (m/z 370.23), C_14_H_16_N_2_O_3_ (m/z 260.12), C_23_H_48_O_8_ (m/z 452.34), and C_14_H_16_N_2_O_2_ (m/z 244.13) based on analysis with the DataAnalysis 4.0 (Bruker Daltonik, Bremen, Germany). 

### 2.6. Genomic Characteristics of A. niger RAF106

**Figure 6:**
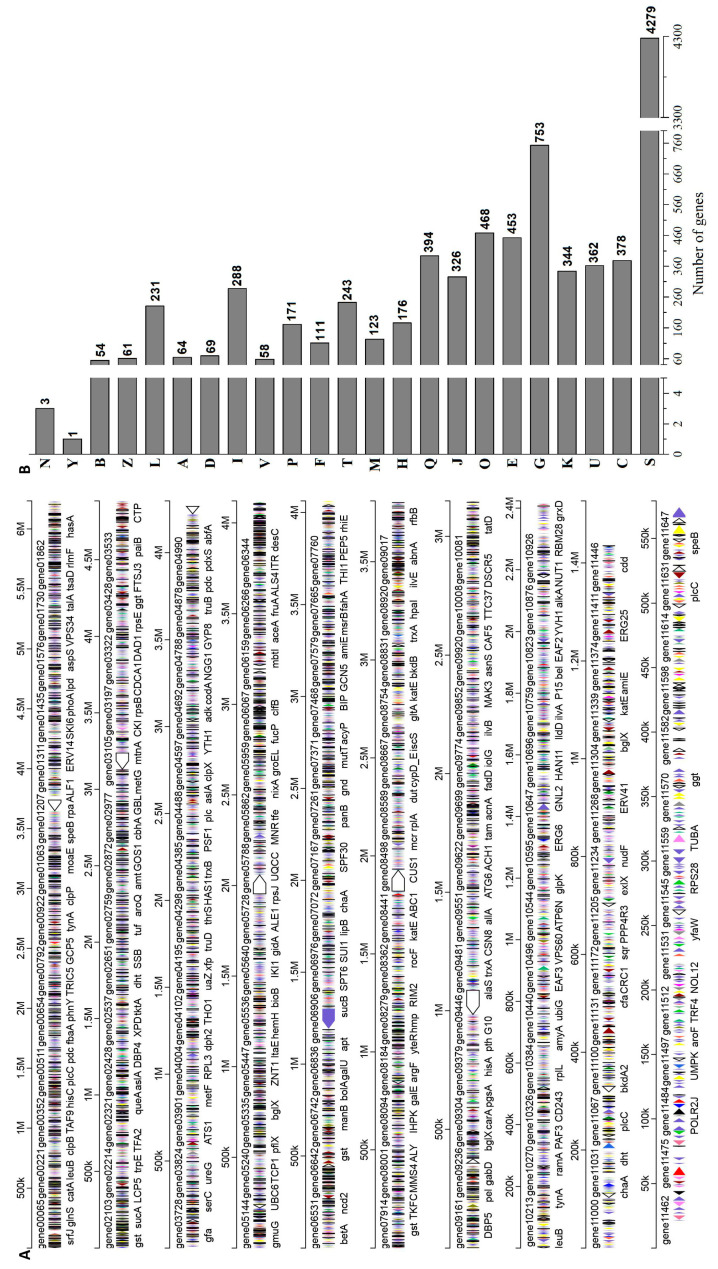
Genome map of *A. niger* RAF106 (**A**) and the clusters of orthologous groups (COG) classification of the genes in the genome (**B**). The letters represent the following COG classifications: C—energy production and conversion; U—intracellular trafficking, secretion, and vesicular transport; S—function unknown; K—transcription; G—carbohydrate transport and metabolism; E—amino acid transport and metabolism; O—post-translational modification, protein turnover, and chaperones; J—translation, ribosomal structure, and biogenesis; Q—secondary metabolites biosynthesis, transport, and catabolism; H—coenzyme transport and metabolism; M—cell wall/membrane/envelope biogenesis; T—signal transduction mechanisms; F—nucleotide transport and metabolism; P—inorganic ion transport and metabolism; V—defense mechanisms; I—lipid transport and metabolism; D—cell cycle control, cell division, and chromosome partitioning; A—RNA processing and modification; L—replication, recombination, and repair; Z—cytoskeleton; B—chromatin structure and dynamics; Y—nuclear structure; N—cell motility.

The genome sequences of *A. niger* RAF106 were obtained using Illumina Hiseq (San Diego, CA, USA) and a PacBio system (Pacific Biosciences, Menlo Park, CA, USA). The assembled genome with a size of 35.09 Mb and a GC content of 49.11% consisted of 10 contigs, 11,323 predicted genes, and 277 tRNAs (Figure 6A). The clusters of orthologous groups (COG) classification showed that 9175 genes were classified into four categories, including metabolism, cellular processes and signaling, information storage and processing, and function unknown (Figure 6B).

Some *A. niger* strains can produce mycotoxins, such as ochratoxin and fumonisin [30]; therefore, secondary metabolic gene clusters were analyzed using antiSMASH (Version 5.0.0, https://antismash.secondarymetabolites.org.) to investigate whether some mycotoxins might be synthesized in *A. niger* RAF106. Here, 88 secondary metabolic gene clusters were identified via an antiSMASH analysis, but only nine gene clusters harbored a 100% similarity to those of known secondary metabolites (Table 1). The gene clusters of mycotoxins, such as fumonisin and ochratoxin, were not found in the *A. niger* RAF106 genome. Moreover, the gene clusters of bioactive compounds, such as pyranonigrin E, TAN-1612, wortmanamide A, HC-toxin, clavaric acid, naphthopyrone, and aureobasidin A1, were found.

Additionally, potential fungal virulence factors were BLASTed in the database of fungal virulence factors (DFVF) using Diamond (Version 0.8.35, https://github.com/bbuchfink/diamond). A total of 59 genes with a similarity of up to 80% were found; however, they were not virulence genes but rather regulatory genes that played essential roles in the regulation of fungal biological processes, including virulence in other fungi (Table 2).

All the data indicated that *A. niger* RAF106 produces beneficial substances but not mycotoxins and could serve as a beneficial and safe fungus to be applied in the processing of food and feed.

## 3. Discussion

Many *A. niger* strains are used extensively in food fermentation and the large-scale production of enzymes, organic acids, and bioactive compounds, which are also used in food and feed processing [24]. In addition, some *A. niger* strains, such as *A. niger* FS-Z1 and foodstuff-derived *A. niger* ND-1, could degrade AFB1 in a time-dependent manner [26,27]. Our results indicated that the tea-derived *A. niger* RAF106, with the exception of converting tea polyphenols into small molecules with high bioavailability [31], could also serve as a safe fungus to efficiently detoxify AFB1 time-dependently at ranges from 0.1 to 4 μg/mL over 72 h in food and feed processing, as discussed below.

First, the AFB1 degradation activity of *A. niger* RAF106 at a similar content of AFB1 and the same fermentation time was significantly higher than that in *A. niger* FS-Z1 and ND-1 [26,27]. Moreover, the hyphal growth of *A. niger* RAF106 was almost unaffected by AFB1 during the degradation of AFB1, which was consistent with the finding that the presence of AFB1 did not appear to inhibit the vegetative growth of any *Pleurotus ostreatus* strains [32]. Additionally, asexual conidiation promotes fungal dispersion. The decrease in the production of conidia caused by AFB1 could suppress the dispersion of *A. niger* RAF106 into other environments during AFB1 degradation.

Moreover, the efficiency of AFB1 degradation mediated by *A. niger* RAF106 was affected by nutrients and fermentation temperatures but not initial pH values. First, the acceleration of AFB1 degradation caused by yeast extract was consistent with that in the AFB1 degradation mediated by *Myroides odoratimimus* 3J2MO, but the inhibition of AFB1 degradation caused by sucrose and lactose, compared with glucose, is opposite to the phenomenon in *M. odoratimimus* 3J2MO and *A. niger* ND-1 [26,33]. Interestingly, the percentage of AFB1 degradation in the presence of yeast extract is similar to and even higher than that of most of the AFB1-degrading bacteria [34]. Changes in the AFB1 degradation caused by different nitrogen sources might have been due to the effects of different nitrogen sources on fungal growth. However, changes in the AFB1 degradation caused by lactose and sucrose differed from their effects on the fungal growth, suggesting that there were other regulators, such as the expression of biosynthetic genes and the enzyme production caused by nitrogen and carbon sources [31,35], that were involved in the regulation of AFB1 degradation in *A. niger* RAF106. Second, the optimal AFB1 degradation for *A. niger* RAF106 was also observed in the temperature range of 25–40 °C, where the percentages of AFB1 degradation were similar to the degradation rates observed in *Bacillus subtilis* UTBSP1 and *Bacillus velezensis* DY3108 [21,36]. The lower AFB1 degradation activities at 25 °C than those at 30–40 °C might be attributed to the decrease in fungal growth at 25 °C following 24 h of incubation. Moreover, increased temperatures may have promoted the bioavailability of organic compounds and facilitated their biodegradation [37], which might also explain the lower AFB1 degradation activities at 25 °C. The lack of a statistically significant difference in the range of 30–40 °C is similar to the findings of Guan et al. [38], Mwakinyali et al. [33], and Shu et al. [21], where they pointed out that the degradation rates of AFB1 demonstrated no significant difference between 20 and 30 °C in *Stenotrophomonas maltophilia* 35-3, between 34 and 37 °C in *M. odoratimimus* 3J2MO, and in the range of 28–30 °C in *B. velezensis* DY3108. Furthermore, the optimal fungal growth for *A. niger* RAF106 was also observed in the temperature range of 25–40 °C, which is in agreement with the previous reports on *A. niger* A-75 and *A. niger* ITAL 704 [39,40]. Additionally, the initial pH value is an important factor in fungal morphology, biomass accumulation, and metabolite formation [41,42]. In this study, the differences between the AFB1 degradation activities and the hyphal biomass at pH values of 4–8 were not significant. The similar AFB1 degradation among different initial pH values ranging from 4 to 8 was similar to the findings of *A. niger* ND-1 in which no significant differences between the percentage of AFB1 degradation at pH values of 6.5–8.0 were observed [26], but the phenomenon differed from the maximum degradation of AFB1 observed at pH 8.5 and higher in *M. odoratimimus* 3J2MO, at pH 8 for *B. velezensis* DY3108, at pH 6.0 for *Rhodicoccus erythropolis*, and between pH 6.5 and 7.5 for *Myxococcus fulvus* ANSM068 [21,33,43,44].

Furthermore, adsorption and degradation are the two primary approaches in the biocontrol of mycotoxin contamination [45]. In *A. niger* RAF106, the AFB1 degradation was mainly due to the enzymatic action of intracellular extracts, which is similar to that reported in *Tetragenococcus halophilus* CGMCC 3792, *Candida versatilis* CGMCC 3790, and *Bacillus licheniformis* BL010, but was opposite to that in *A. niger* ND-1, where AFB1 degradation was mainly caused by the involvement of enzymes from extracellular extracts [26,46,47,48]. In *A. niger* ND-1, heat treatment dramatically decreased the AFB1 degradation activities of the culture supernatant [26]. Interestingly, the percentage of AFB1 degradation mediated by intracellular extracts in this study remained at more than 50% after the intracellular extracts were boiled for 20 min. The remaining AFB1 degradation activities were similar to those in *Escherichia coli* CG1061 but higher than those in *C. versatilis* CGMCC 3790, *B. subtilis* UTBSP1, and *T. halophilus* CGMCC 3792 [36,46,47,49]. These results suggested that intracellular enzymes or proteins with excellent thermostability were involved in the AFB1 degradation in *A. niger* RAF106.

Lastly, the process of using *A. niger* RAF106 to degrade AFB1 was safe. For one thing, the degradation products had no mutagenicity according to the Ames test, which was consistent with the findings in *Aspergillus oryzae* MAO103 and MAO104, *B. licheniformis* CFR1 and *R. erythropolis* [13,50,51]. It has been reported that the mutagenic effects of AFB1 were caused by both the lactone portion and the dihydrofuran moiety of the molecule [6,7,13]. The decreases in mutagenicity suggested that *A. niger* RAF106 might detoxify AFB1 into less toxic or non-toxic compounds without the lactone portion and/or the dihydrofuran moiety. For another thing, the genome data indicated that *A. niger* RAF106 does not produce mycotoxins or contain virulence genes. The absence of gene clusters of mycotoxins, such as fumonisin and ochratoxin in the *A. niger* RAF106 genome differed from the findings in *A. niger* NRRL 3122 and CBS 513.88 [29,52]. Moreover, the predicted metabolites from the genome of *A. niger* RAF106 showed the presence of pyranonigrin E, TAN-1612, wortmanamide A, HC-toxin, clavaric acid, naphthopyrone, and aureobasidin A1, which are active substances with anticancer, antimutagenic, antifungal, and/or antioxidant activities [53,54,55,56,57,58]. In addition, the genome also contained the genes for 12 cellulases, seven pectinases, and nine amylases (data not shown), which degrade complex polysaccharides and are important and widely used in food processing [25]. The size of the genome and the GC content are similar to those of *A. niger* L2 (36.45 Mb and 49.20%, respectively), *A. niger* H915 (35.98 Mb and 49.20%, respectively), *A. niger* An76 (34.88 Mb and 49.40%, respectively), *A. niger* SH2 (34.63 Mb and 50.26% respectively), and *A. niger* CBS 513.88 (34.02 Mb and 50.04%, respectively) [29,59,60,61].

Overall, *A. niger* RAF106 and its intracellular extracts could serve as beneficial and safe bio-carriers to effectively detoxify AFB1 and are suitable for the various food and feed processing environments.

## 4. Conclusions

*Aspergillus niger* RAF106 could tolerate AFB1 and effectively detoxify it to compounds with less mutagenicity in environments with pH values from 4 to 10 and temperatures from 25 to 45 °C. More importantly, to the best of our knowledge, this is the first study to demonstrate more than 80% degradation of AFB1 over 24 h of incubation in *A. niger* strains. The degradation was attributed to intracellular proteins or enzymes with excellent thermostability. Moreover, a genomic analysis indicated that *A. niger* RAF106 is a safe and potentially useful fungus that lacks virulence genes and does not synthesize mycotoxins. However, further studies are needed to illuminate the structures of the degradation products and the enzymatic characteristics of the intracellular enzymes. In summary, *A. niger* RAF106 could potentially be a safe, beneficial, and commercial fungus to be applied to detoxify AFB1 in food and feed processing and industries.

## 5. Material and Methods

### 5.1. Fungal Strains and Culture Conditions

*Aspergillus niger* RAF106 (CGMCC NO.9608), the catechin-transforming fungus isolated from Pu-erh tea [31], was cultivated in potato dextrose agar (PDA) at 30 °C to produce conidia, Sabouraud dextrose broth (1% tryptone and 4% glucose, pH 7.0) to degrade AFB1, and SDA (1% tryptone, 4% glucose, and 2% agar, pH 7.0) to detect the effect of AFB1 on the conidiation capacity. The conidia were harvested by washing the mature culture on the medium with sterilized 0.02% Tween 80. *Salmonella typhimurium* TA98 and TA100 purchased from Moltox (Boone, NC, USA) were cultivated in beef extract peptone medium at 37 °C for the Ames test.

### 5.2. AFB1 Degradation by A. niger RAF106 in Liquid Culture

A suspension of 500 μL of 10^8^ conidia/mL (the same is used below unless specified) was incubated in 100 mL SDB supplemented with gradient concentrations of AFB1 (0.1–4 μg/mL) purchased from J&K Scientific (Beijing, China) for 3 days at 30 °C via shaking at 180 rpm. During 3 days of incubation with shaking at 30 °C, sterile SDB with gradient concentrations of AFB1 was used as the control. The supernatant was extracted daily using half the volume of chloroform three times by shaking at 180 rpm, as previously described [38,46,49], and the chloroform fractions were evaporated and dissolved using dimethyl sulfoxide (DMSO, Sigma-Aldrich, St. Louis, MO, USA). The redissolved solution was filtered through a 0.22 μm pore filter (Merck-Millipore, Darmstadt, Germany) and stored at −20 °C for HPLC detection. Using chloroform extraction, 94–96% of the AFB1 was recovered from the liquid culture. All of the experiments were conducted in triplicate.

### 5.3. Quantification of AFB1 Using HPLC 

AFB1 and the subsequent degradation products were analyzed using HPLC (Waters Alliance 2695, Milford, MA, USA) equipped with a YMC C18 column (250 × 4.6 mm, 5 μm) and a UV detector set at 360 nm [62]. A volume of 10 μL of samples was injected and eluted with the mobile solvent consisting of water/methanol/acetonitrile (48:40:12, v/v/v) at a constant flow rate of 0.8 mL/min at 30 °C. The limit of detection (LOD) was 0.01 μg/mL AFB1 and the limit of quantitation was 0.05 μg/mL AFB1 under the experimental conditions used. The concentration of AFB1 was determined using external calibration curves of the standard AFB1 solution at different concentrations. The percentage of AFB1 degradation was calculated using the following formula: AFB1 degradation (%) = (1 − remaining AFB1 in sample/total AFB1 in control sample) × 100.

### 5.4. Changes in Fungal Growth and Aerial Conidiation Caused by AFB1 in A. niger RAF106 

The fungal growth was measured first by assessing the fungal biomass when aliquots of conidial suspension were incubated in SDB and SDB with 2 μg/mL AFB1 for 3 days at 30 °C while shaking at 180 rpm. Briefly, the hyphae were collected daily, dried at 60 °C, and weighed. The data was converted to the weight per mL culture. Second, aliquots of 1 μL of a 10^6^ conidia/mL suspension were spotted centrally onto plates (60 mm diameter) of SDA and SDA supplemented with 2 μg/mL AFB1 at 30 °C. After 3 days, the diameter of each colony was estimated using two measurements taken perpendicular to each other across the center of the colony. All of the experiments were conducted in triplicate. 

The aerial conidiation capacity was quantified by spreading 50 μL of a 10^7^ conidia/mL suspension on SDA plates (60 mm diameter) and SDA plates with 2 μg/mL AFB1 followed by 3 days of cultivation at 30 °C. On days 1, 2, and 3, all the conidia from each plate were washed and harvested with 5 mL of 0.02% Tween 80 using vibration. The concentration of conidia in the suspension was determined using a hemocytometer and converted to the number of conidia per square centimeter of plate culture. All of the experiments were conducted in triplicate.

### 5.5. Effects of Nutrients, pH, and Temperature on AFB1 Degradation and Fungal Growth

The effects of the nutrients on AFB1 degradation were determined in modified SDB. The modified media were prepared by replacing tryptone in SDB with yeast extract, NaNO_3_, or NaNO_2_ as the single nitrogen source and replacing glucose with lactose or sucrose as the single carbon source. The effects of pH and temperature were determined in modified SDB with yeast extract by adjusting the initial pH values to 4.0, 5.0, 6.0, 7.0, 8.0, or 10.0 and by setting the initial temperature to 25, 30, 37, 40, or 45 °C. Aliquots of the conidial suspensions were added to the modified SDB with 2 μg/mL AFB1 and incubated for 72 h with constant shaking (180 rpm). Correspondingly, the modified SDB with 2 μg/mL AFB1 in each incubation was used as the control. The residual AFB1 was determined using HPLC analysis. In addition, the fungal growth was also investigated by measuring the biomass. All of the experiments were conducted in triplicate.

### 5.6. AFB1 Degradation by Extracellular Extracts, Intracellular Extracts, and Dead Cells

Aliquots of conidial suspensions were pre-cultivated in SDB at 30 °C with shaking at 180 rpm for 48 h. After centrifugation at 12,000 rpm at 4 °C for 15 min, the supernatant and cells were collected. The supernatant was filtered with a 0.22 μm pore filter and served as the extracellular extracts for the degradation of AFB1. The cells were washed three times with a 10 mM phosphate buffer (pH 7.4), ground in liquid nitrogen, suspended in an equal volume of a 10 mM phosphate buffer (pH 7.4), and centrifuged for 20 min at 12,000 rpm at 4 °C. The supernatant was filtered with the 0.22 μm pore filter and served as the intracellular extracts for the degradation of AFB1. Moreover, the cells that had been washed three times were boiled for 20 min, resuspended in an equal volume of 10 mM phosphate buffer (pH 7.4), and served as the dead cells for the degradation of AFB1. The extracellular extracts, intracellular extracts, and dead cells were incubated with 2 μg/mL AFB1 at 30 °C with shaking at 180 rpm. Non-incubated cultures (SDB for extracellular extracts and phosphate buffer for intracellular extracts and dead cells) supplemented with 2 μg/mL AFB1 were used as the control, and all the variables in the control groups were similar to those for the corresponding extracts and dead cells. At 24 h intervals, the residual AFB1 was tested as described above. All of the experiments were conducted in triplicate.

### 5.7. Effects of Proteinase K, SDS, and Heat Treatment on the Degradation of AFB1 

The effects of proteinase K (a serine protease, purchased from Sangon, Shanghai, China), SDS (purchased from Fuyu Fine Chemical Co., Ltd., Tianjin, China), and heat treatment on AFB1 degradation were performed to investigate whether the degradation of AFB1 was mediated by thermostable enzymes or proteins in *A. niger* RAF106. The intracellular extracts were boiled for 20 min and treated with 1 mg/mL proteinase K, 1% SDS, or SDS plus proteinase K for 6 h at 30 °C. Subsequently, each sample was incubated with 2 μg/mL AFB1 at 30 °C with constant shaking (180 rpm), and phosphate buffer with 2 μg/mL AFB1 was used as the control. After 48 h, the residue of AFB1 was monitored as described above. All of the experiments were conducted in triplicate.

### 5.8. Analysis of the AFB1 Metabolites Using HPLC-Q-TOF-MS

After a 72-h incubation of *A. niger* RAF106 in SDB with 2 μg/mL AFB1, the metabolites were extracted with chloroform as described above and separated using an Agilent 1260 series HPLC system equipped with an auto-injector and quaternary HPLC pump (Agilent Technologies, Santa Clara, CA, USA) [38,46,49]. The chromatography was conducted on a Phenomenex Luna 5u C18(2)100A column (250 × 4.60 mm, 5 μm) (Torrance, CA, USA). Samples (10 μL) were injected and eluted with the mobile solvent that contained 45:55 of methanol and water (v/v) with 0.1% formic acid. The total run time was 30 min with a flow rate of 0.5 mL/min. An MS analysis was performed with a Bruker maXis ESI Q-TOF (Bruker Daltonik, Bremen, Germany). The acquisition parameters were as follows: the capillary voltage was set at 4.5 kV in the positive ionization mode and the dry temperature was 180 °C. The drying gas flowed at 5.0 L/min and the nebulizer was at 0.8 bar. The MS was operated in full scan mode, and the data were collected within the range of m/z 100–2000. Samples extracted from SDB with 2 μg/mL AFB1 were used as the positive control and samples extracted from SDB and cultures of *A. niger* RAF106 in SDB were used as the negative control.

### 5.9. Ames Mutagenicity Assay

The *Salmonella* (Ames) test with metabolic activation was used as a proxy to evaluate the mutagenicity of the degradation products [63]. The Ames assay was conducted with the S9 Enzyme Activation Kit (Iphase Pharma Service, Beijing, China) that is based on the validated Ames bacterial reverse mutation assay, according to the manufacturer’s instructions. Briefly, the degradation products extracted from a 72 h culture co-incubated with *A. niger* RAF106 and AFB1 were incubated with *S. typhimurium* TA98 or TA100 at 37 °C. After 48 h, the number of *S. typhimurium* colonies was recorded, and the data were shown as the number of revertant CFUs. A sample extracted from SDB with AFB1 was used as the positive control and extracts from SDB were used as the negative control. The manufacturer’s instructions indicated that the revertant CFUs must be controlled within the range from 150 to 250; therefore, the dose-dependent curve of the revertant CFUs caused by AFB1 was first established. A concentration of 20 ng/plate of AFB1 was chosen as the mutagenic dose based on the curve. All of the experiments were conducted in triplicate.

### 5.10. Genome Sequencing and Analysis

Genomic DNA was extracted using an Omega Fungal DNA Kit D3390-02 (Qiagen, Hilden, Germany) according to the manufacturer’s instructions, and genome sequencing was performed with a combination of the PacBio Sequel Single-Molecule Real-Time (SMRT) (Pacific Biosciences, Menlo Park, CA, USA) and Illumina sequencing platforms (HiSeq X Ten; Illumina, San Diego, CA, USA). After clean data were obtained, the reads were then assembled into contigs using CANU (Version 1.7, http://canu.readthedocs.io/en/latest/). Finally, error correction of the PacBio assembly results was performed using the Illumina reads. The sequence data for *A. niger* RAF106 was deposited at the US National Center for Biotechnology Information (NCBI) under the accession number RIBO00000000. 

Identification of the predicted coding sequences (CDSs) was performed using Maker2 (Version 2.31.9, http://www.yandell-lab.org/software/maker.html), and tRNA-scan-SE (Version 2.0, http://trna.ucsc.edu/software/) was used for tRNA searching. The predicted CDSs were annotated from the databases of NR (Non-Redundant Protein Sequence Database), Swiss-Prot, Pfam, Go Ontology (GO), COG, and Kyoto Encyclopedia of Genes and Genomes (KEGG) using sequence alignment tools, namely, Basic Local Alignment Search Tool (BLAST; Version 2.3.0, ftp://ftp.ncbi.nlm.nih.gov/blast/executables/blast+/2.3.0/), Diamond, and HMMER (Version 3.1b2, http://www.hmmer.org/). Briefly, each set of query proteins were aligned with the databases, and annotations of the best-matched subjects (E-value < 10^−5^) were obtained for the gene annotation. Secondary metabolite clusters were predicted using antiSMASH, and all of the analyses were performed using the I-Sanger Cloud Platform (www.i-sanger.com) from Shanghai Majorbio (Shanghai, China).

### 5.11. Data Analysis

Results from three replicates were expressed as mean ± standard deviation (SD), and the data analysis was subjected to one-factor analysis of variance (ANOVA), followed by Tukey’s HSD test. In addition, Student’s *t*-test and the *F*-test were applied to compare the differences between two samples and between three and more samples, respectively. Results were considered to be statistically significant when * *p* < 0.05 in all experiments.

## Figures and Tables

**Table 1:** Secondary metabolites predicted by the antiSMASH analysis of *A. niger* RAF106.

Cluster Type	MIBiG ID *	Similarity	Cluster Type	MIBiG ID	Similarity
**Scaffold 1**			**Scaffold 2**		
Aflatrem	BGC0000629	11%	Aflavarin	BGC0001304	40%
Notoamide	BGC0000818	11%	Nidulanin A	BGC0001699	75%
Pyripyropene A	BGC0001068	25%	Fusarielin H	BGC0001600	37%
**Scaffold 3**			Pyranonigrin E	BGC0001124	100%
Fumonisin	BGC0000063	11%			
**Scaffold 5**			**Scaffold 6**		
Azaphilone	BGC0000027	16%	HC-toxin	BGC0000370	100%
Pyranonigrin E	BGC0001124	100%	Clavaric acid	BGC0001248	100%
TAN-1612	BGC0000156	100%	Naphthopyrone	BGC0000107	100%
Wortmanamide A	BGC0001466	100%	Aflatrem	BGC0000629	17%
Communesin	BGC0001205	12%			
Shearinine D	BGC0001776	13%	**Scaffold 9**		
**Scaffold 7**			Aureobasidin A1	BGC0000307	100%
Citrinin	BGC0001338	18%	HC-toxin	BGC0000370	100%
Aflatrem	BGC0000629	25%	Duclauxin	BGC0001578	42%
Neurosporin A	BGC0001697	40%			

* MIBiG ID represents the identification number (ID) of clusterin MIBiG (Minimum Information about a Biosynthetic Gene cluster) database. The similarity between the predicted clusters and the clusters in MiBIG is expressed in parentheses.

**Table 2:** Genes with a similarity of up to 80% found in *A. niger* RAF106 according to the database of fungal virulence factors.

Gene ID	Annotation	Similarity
**Scaffold 1**		
gene00348	Secondary metabolism regulator laeA	83.1%
gene00577	40S ribosomal protein S27a	100%
gene00593	Aspartic protease Pep2	87.7%
gene00993	Pyrophosphate phospho-hydrolase	93.1%
gene01168	Cell division control protein 42	80.1%
gene01307	Phosphatidate cytidylyltransferase	88.7%
gene01444	Transcriptional regulatory protein RPD3	87.8%
gene01632	Chitin synthase C	80.9%
gene01911	Fatty acid desaturase	92.8%
**Scaffold 2**		
gene02216	1,3-beta-glucan synthase component FKS1	89%
gene02229	Translation initiation factor eif-2b epsilon subunit	83.4%
gene02256	Mitogen-activated protein kinase	93.4%
gene02307	GTP-binding nuclear protein GSP1/Ran	86%
gene02534	Histone H3	97.7%
gene02609	Guanine nucleotide-binding protein subunit α	94.1%
gene02632	Mitogen-activated protein kinase hog1	95.1%
gene02634	G protein complex alpha subunit GpaB	91.6%
gene02843	β-tubulin	97%
gene03109	Glucosamine-fructose-6-phosphate aminotransferase	96.4%
gene03500	Guanine nucleotide-binding protein subunit β	85.2%
**Scaffold 3**		
gene03862	Bifunctional tryptophan synthase TRPB	85.2%
gene03939	Secretory pathway GDP dissociation inhibitor	84.6%
gene03951	GlcNAc-1-P transferase	81.3%
gene03962	Ribosomal RNA assembly protein mis3	88.3%
gene04302	Mitogen-activated protein kinase Fus3	85.5%
gene04382	Thioredoxin reductase Trr1/Trr2	85.3%
gene04618	α-Tubulin	92.3%
gene04834	Ubiquitin	95.9%
**Scaffold 4**		
gene05492	Tubulin α-1 chain	90.8%
gene05524	Ferrochelatase	87.6%
gene05596	Citrate synthase Cit1	88.4%
gene05603	Malate synthase	81.8%
gene05712	Imidazoleglycerol-phosphate dehydratase	86.9%
gene05752	Mitochondrial import receptor subunit tom40	86.5%
gene05805	Ornithine-N5-oxygenase	81.4%
gene05876	Peptidyl-prolyl cis-trans isomerase ssp-1	81.6%
**Scaffold 5**		
gene07009	Rho-related protein rac1	85.1%
gene07037	Phosphoadenosine phosphosulfate reductase	85.1%
gene07271	U1 snRNP splicing complex subunit	94.6%
gene07607	Cell division control protein 2	90%
**Scaffold 6**		
gene08487	Secretion related GTPase	82.6%
gene08506	GTP-binding protein	99%
gene08568	S-adenosylmethionine decarboxylase proenzyme	83.4%
gene08576	Glutamyl-tRNA synthetase	83.4%
gene08706	Golgi transport protein Sly1	84.4%
**Scaffold 7**		
gene09163	1,3-β-glucanosyltransferase Gel1	85.4%
gene09291	Homoserine O-acetyltransferase	87.2%
gene09449	GTP-binding protein rhb1	95.2%
gene09466	Acetyl-CoA acetyltransferase	92.2%
gene09749	Eukaryotic translation initiation factor 3 subunit	80.6%
gene09874	Aureobasidin resistance protein Aur1	92%
gene09879	Sec14 cytosolic factor	84.8%
gene09963	DNA primase subunit Pri1	80.4%
**Scaffold 8**		
Gene10341	Orotate phosphoribosyltransferase	80.9%
Gene10380	T-complex protein 1	82.8%
Gene10446	Polyubiquitin	98.7%
Gene10497	Ribose-phosphate pyrophosphokinase	86.1%
Gene10549	Saccharopine dehydrogenase	89.5%
Gene10566	Mannose-1-phosphate guanyltransferase	93.7%

## References

[1] Lee H.J., Ryu D. (2017). Worldwide occurrence of mycotoxins in cereals and cereal-derived food products: Public health perspectives of their co-occurrence. J. Agric. Food Chem..

[2] Eskola M., Kos G., Elliott C.T., Hajšlová J., Mayar S., Krska R. (2019). Worldwide contamination of food-crops with mycotoxins: Validity of the widely cited ‘FAO estimate’ of 25%. Crit. Rev. Food Sci..

[3] Nugraha A., Khotimah K., Rietjens I.M.C.M. (2018). Risk assessment of aflatoxin B_1_ exposure from maize and peanut consumption in Indonesia using the margin of exposure and liver cancer risk estimation approaches. Food Chem. Toxicol..

[4] Cui A., Hua H., Shao T., Song P., Kong Q., Luo T., Jiang Y. (2015). Aflatoxin B_1_ induces Src phosphorylation and stimulates lung cancer cell migration. Tumour Biol..

[5] Lahoum A., Verheecke-Vawssen C., Bouras N., Sabaou N., Mathieu F. (2017). Taxonomy of mycelial actinobacteria isolated from Saharan soils and their effiency to reduce aflatoxin B_1_ content in a solid-based medium. Ann. Microbiol..

[6] Lee L.S., Dunn J.J., Delucca A.J., Ciegler A. (1981). Role of lactone ring of aflatoxin B_1_ in toxicity and mutagenicity. Experientia.

[7] Wogan G., Edwards G., Newberne P. (1971). Structure-activity relationships in toxicity and carcinogenicity of aflatoxins and analogs. Cancer Res..

[8] Cullen J.M., Ruebner B.H., Hsieh L.S., Hyde D.M., Hsieh D.P. (1987). Carcinogenicity of dietary aflatoxin M_1_ in male fischer rats compared to aflatoxin B_1_. Cancer Res..

[9] Luongo D., Russo R., Balestrieri A., Marzocco S., Bergamo P., Severino L. (2013). In vitro study of AFB_1_ and AFM_1_ effects on human lymphoblastoid Jurkat T-cell model. J. Immunotoxicol..

[10] Pitt J.I., Miller J.D. (2017). A concise history of mycotoxin research. J. Agric. Chem..

[11] Wu F., Mitchell N.J. (2016). How climate change and regulations can affect the economics of mycotoxins. World Mycotoxin J..

[12] Williams J.H., Phillips T.D., Jolly P.E., Stiles J.K., Jolly C.M., Aggarwal D. (2004). Human aflatoxicosis in developing countries: A review of toxicology, exposure, potential health consequences, and interventions. Am. J. Clin. Nutr..

[13] Lee K.R., Yang S.M., Cho S.M., Kim M., Hong S.Y., Chung S.H. (2017). Aflatoxin B_1_ detoxification by *Aspergillus oryzae* from Meju, a traditional Korean fermented soybean starter. J. Microbiol. Biotechnol..

[14] Mishra H.N., Das C. (2003). A review on biological control and metabolism of aflatoxin. Front. Pharmacol..

[15] Wu Q., Jezkova A., Yuan Z., Pavlikova L., Dohnal V., Kuca K. (2009). Biological degradation of aflatoxins. Drug Metab. Rev..

[16] Chen Y., Kong Q., Chi C., Shan S., Guan B. (2015). Biotransformation of aflatoxin B-1 and aflatoxin G(1) in peanut meal by anaerobic solid fermentation of *Streptococcus thermophilus* and *Lactobacillus delbrueckii* subsp. bulgaricus. Int. J. Food Microbiol..

[17] Samuel M.S., Sivaramakrishna A., Mehta A. (2014). Degradation and detoxification of aflatoxin B_1_ by *Pseudomonas putida*. Int. Biodeter. Biodegrad..

[18] Eshelli M., Harvey L., Edrada-Ebel R., McNeil B. (2015). Metabolomics of the bio-degradation process of aflatoxin B_1_ by actinomycetes at an initial pH of 6.0. Toxins.

[19] Wang J., Ogata M., Hirai H., Kawagishi H. (2011). Detoxification of aflatoxin B_1_ by manganese peroxidase from the white-rot fungus *Phanerochaete sordida* YK-624. FEMS Microbiol. Lett..

[20] Ji C., Fan Y., Zhao L. (2016). Review on biological degradation of mycotoxins. Anim. Nutr..

[21] Shu X., Wang Y., Zhou Q., Li M., Hu H., Ma Y., Chen X., Ni J., Zhao W., Huang S. (2018). Biological degradation of Aflatoxin B-1 by cell-free extracts of *Bacillus velezensis* DY3108 with broad pH stability and excellent thermostability. Toxins.

[22] Adebo O.A., Njobeh P.B., Sidu S., Tlou M.G., Mavumengwana V. (2016). Aflatoxin B_1_ degradation by liquid cultures and lysates of three bacterial strains. Int. J. Food Microbiol..

[23] Cairns T.C., Nai C., Meyer V. (2018). How a fungus shapes biotechnology: 100 years of *Aspergillus niger* research. Fungal Biol. Biotechnol..

[24] Park H.S., Jun S.C., Han K.H., Hong S.B., Yu J.H. (2017). Diversity, application, and synthetic biology of industrially important *Aspergillus fungi*. Adv. Appl. Microbiol..

[25] Ma Y., Ling T., Su X., Jiang B., Nian B., Chen L., Liu M., Zhang Z., Wang D., Mu Y. (2020). Integrated proteomics and metabolomics analysis of tea leaves fermented by *Aspergillus niger*, *Aspergillus tamarii* and *Aspergillus fumigatus*. Food Chem..

[26] Zhang W., Xue B., Li M., Mu Y., Shan A. (2014). Screening a strain of *Aspergillus niger* and optimization of fermentation conditions for degradation of Aflatoxin B_1_. Toxins.

[27] Sun X., Sun C., Zhang X., Zhang H., Tang L. (2015). Aflatoxin B_1_ decontamination by UV-mutated live and immobilized *Aspergillus niger*. Food Control.

[28] Mann R., Rehm H.J. (1976). Degradation products from aflatoxin B_1_ by *Corynebacterium rubrum*, *Aspergillus niger*, *Trichoderma viride* and *Mucor ambiguus*. Appl. Microbiol. Biotechnol..

[29] Andersen M.R., Salazar M.P., Schaap P.J., Van de Vondervoort P.J.I., Culley D., Thykaer J., Frisvad J.C., Nielsen K.F., Albang R., Albermann K. (2011). Comparative genomics of citric-acid-producing *Aspergillus niger* ATCC 1015 versus enzyme-producing CBS 513.88. Genome Res..

[30] Frisvad J.C., Møller L.L.H., Larsen T.O., Kumar R., Arnau J. (2018). Safety of the fungal workhorses of industrial biotechnology: Update on the mycotoxin and secondary metabolite potential of *Aspergillus niger*, *Aspergillus oryzae*, and *Trichoderma reesei*. Appl. Microbiol. Biotechnol..

[31] Fang X., Du M., Liu T., Fang Q., Liao Z., Zhong Q., Chen J., Meng X., Zhou S., Wang J. (2019). Changes in the biotransformation of green tea catechins induced by different carbon and nitrogen sources in *Aspergillus niger* RAF106. Front. Microbiol..

[32] Jackson L.W., Pryor B.M. (2017). Degradation of aflatoxin B_1_ from naturally contaminated maize using the edible fungus *Pleurotus ostreatus*. AMB Express.

[33] Mwakinyali S.E., Ming Z., Xie H., Zhang Q., Li P. (2019). Investigation and characterization of *Myroides odoratimimus* Strain 3J2MO aflatoxin B_1_ degradation. J. Agric. Food Chem..

[34] Verheecke C., Liboz T., Mathieu F. (2016). Microbial degradation of aflatoxin B_1_: Current status and future advances. Int. J. Food Microbiol..

[35] Brzonkalik K., Herrling T., Syldatk C., Neumann A. (2011). The influence of different nitrogen and carbon sources on mycotoxin production in *Alternaria alternate*. Int. J. Food Microbiol..

[36] Farzaneh M., Shi Z., Ghassempour A., Sedaghat N., Ahmadzadeh M., Mirabolfathy M., Javan-Nikkhah M. (2012). Aflatoxin B_1_ degradation by *Bacillus subtilis* UTBSP1 isolated from pistachio nuts of Iran. Food Control.

[37] Müller R., Antranikian G., Maloney S., Sharp R. (2007). Thermophilic degradation of environmental pollutants. In: Biotechnology of Extremophiles. Adv. Biochem. Eng. Biotechnol..

[38] Guan S., Cheng J., Ting Z., Junxia L., Qiugang M., Tiangui N. (2008). Aflatoxin B_1_ degradation by *Stenotrophomonas Maltophilia* and other microbes selected using coumarin medium. Int. J. Mol. Sci..

[39] Alborch L., Bragulat M.R., Abarca M.L., Cabañes F.J. (2011). Effect of water activity, temperature and incubation time on growth and ochratoxin a production by *Aspergillus niger* and *Aspergillus carbonarius* on maize kernels. Int. J. Food Microbiol..

[40] Héctor P., Hiromi T.M., Minoru H.J., De M.H.C. (2005). Growth of *Aspergillus ochraceus*, *A. carbonarius* and *A. niger* on culture media at different water activities and temperatures. Braz. J. Microbiol..

[41] Colin V., Baigorí M., Pera L. (2013). Tailoring fungal morphology of *Aspergillus niger* MYA 135 by altering the hyphal morphology and the conidia adhesion capacity: Biotechnological applications. AMB Express.

[42] Shu C.H., Lung M.Y. (2004). Effect of pH on the production and molecular weight distribution of exopolysaccharide by *Antrodia* camphorate in batch cultures. Process Biochem..

[43] Kong Q., Zhai C., Guan B., Li C., Shan S., Yu J. (2012). Mathematic modeling for optimum conditions on aflatoxin B_1_ degradation by the aerobic bacterium *Rhodococcus erythropolis*. Toxins.

[44] Zhao L.H., Guan S., Gao X., Ma Q.G., Lei Y.P., Bai X.M., Ji C. (2010). Preparation, purification and characteristics of an aflatoxin degradation enzyme from *Myxococcus fulvus* ANSM068. J. Appl. Microbiol..

[45] Hathout A.S., Aly S.E. (2014). Biological detoxification of mycotoxins: A review. Ann. Microbiol..

[46] Li J., Huang J., Jin Y., Wu C., Shen D., Zhang S., Zhou R. (2018). Aflatoxin B_1_ degradation by salt tolerant *Tetragenococcus halophilus* CGMCC 3792. Food Chem. Toxicol..

[47] Li J., Huang J., Jin Y., Wu C., Shen D., Zhang S., Zhou R. (2018). Mechanism and kinetics of degrading aflatoxin B_1_ by salt tolerant *Candida versatilis* CGMCC 3790. J. Hazard. Mater..

[48] Wang Y., Zhang H., Yan H., Yin C., Liu Y., Xu Q., Liu X., Zhang Z. (2018). Effective biodegradation of aflatoxin B_1_ using the *Bacillus licheniformis* (BL010) strain. Toxins.

[49] Wang L., Wu J., Liu Z., Shi Y., Liu J., Xu X., Hao S., Mu P., Deng F., Deng Y. (2019). Aflatoxin B_1_ degradation and detoxification by *Escherichia coli* CG1061 isolated from chicken. Front. Pharmacol..

[50] Rao K.R., Vipin A.V., Hariprasad P., Appaiah K.A.A., Venkateswaran G. (2016). Biological detoxification of aflatoxin B_1_ by *Bacillus licheniformis* CFR1. Food Control.

[51] Alberts J.F., Engelbrecht Y., Steyn P.S., Holzapfel W.H., Van Zyl W.H. (2006). Biological degradation of aflatoxin B_1_ by *Rhodococcus erythropolis* cultures. Int. J. Food Microbiol..

[52] Pel H.J., De Winde J.H., Archer D.B., Dyer P.S., Hofmann G., Schaap P.J., Turner G., De Vries R.P., Albang R., Albermann K. (2007). Genome sequencing and analysis of the versatile cell factory *Aspergillus niger* CBS 513.88. Nat. Biotechnol..

[53] Miyake Y., Ito C., Itoigawa M., Osawa T. (2007). Isolation of the antioxidant pyranonigrin-A from rice mold starters used in the manufacturing process of fermented foods. J. Agric. Chem. Soc. Jpn..

[54] Inglis D.O., Binkley J., Skrzypek M.S., Arnaud M.B., Cerqueira G.C., Shah P., Wymore F., Wortman J.R., Sherlock G. (2013). Comprehensive annotation of secondary metabolite biosynthetic genes and gene clusters of *Aspergillus nidulans*, *A. fumigatus*, *A. niger* and *A. oryzae*. BMC Microbiol..

[55] Kurome T., Inami K., Inoue T., Ikai K., Takesako K., Kato I., Shiba T. (1996). Total synthesis of an antifungal cyclic depsipeptide aureobasidin A. Tetrahedron.

[56] Choi J.S., Lee H.J., Park K.Y., Ha J.O., Kang S.S. (1997). In vitro antimutagenic effects of anthraquinone aglycones and naphthopyrone glycosides from *Cassia tora*. Planta Med..

[57] Walton J.D. (2006). HC-toxin. Phytochemistry.

[58] Godio R.P., Fouces R., Gudiña E.J., Martín J.F. (2004). *Agrobacterium tumefaciens*-mediated transformation of the antitumor clavaric acid-producing basidiomycete *Hypholoma sublateritium*. Curr. Genet..

[59] Yin C., Wang B., He P., Lin Y., Pan L. (2014). Genomic analysis of the aconidial and high-performance protein producer, industrially relevant *Aspergillus niger* SH_2_ strain. Gene.

[60] Gong W., Cheng Z., Zhang H., Liu L., Gao P., Wang L. (2016). Draft genome sequence of *Aspergillus niger* strain An76. Genome Announc..

[61] Yin X., Shin H., Li J., Du G., Liu L., Chen J. (2017). Comparative genomics and transcriptome analysis of *Aspergillus niger* and metabolic engineering for citrate production. Sci. Rep..

[62] Malekpour A., Bayati S. (2015). Stimultaneous determination of aflatoxins in pistachio using ultrasonically stabilized chloroform/water emulsion and HPLC. Food Anal. Methods.

[63] Ames B.N., Durston W.E., Yamasaki E., Lee F.D. (1973). Carcinogens are mutagens: A simple test system combining liver homogenates for activation and bacteria for detection. Proc. Natl. Acad. Sci. USA.

